# PEGylation Extends Circulation Half-Life While Preserving *In Vitro* and *In Vivo* Activity of Tissue Inhibitor of Metalloproteinases-1 (TIMP-1)

**DOI:** 10.1371/journal.pone.0050028

**Published:** 2012-11-20

**Authors:** Jyotica Batra, Jessica Robinson, Christine Mehner, Alexandra Hockla, Erin Miller, Derek C. Radisky, Evette S. Radisky

**Affiliations:** Department of Cancer Biology, Mayo Clinic Cancer Center, Jacksonville, Florida, United States of America; University of Patras, Greece

## Abstract

Excess proteolytic activity of matrix metalloproteinases (MMPs) contributes to the development of arthritis, cardiovascular diseases and cancer progression, implicating these enzymes as therapeutic targets. While many small molecule inhibitors of MMPs have been developed, clinical uses have been limited, in part by toxicity and off-target effects. Development of the endogenous tissue inhibitors of metalloproteinases (TIMPs) as recombinant biopharmaceuticals represents an alternative therapeutic approach; however, the short plasma half-life of recombinant TIMPs has restricted their potential in this arena. To overcome this limitation, we have modified recombinant human TIMP-1 (rhTIMP-1) by PEGylation on lysine residues. We analyzed a mixture of mono- and di-PEGylated rhTIMP-1 species modified by attachment of 20 kDa mPEG chains (PEG_20K_-TIMP-1), as confirmed by SELDI-TOF mass spectrometry. This preparation retained complete inhibitory activity toward the MMP-3 catalytic domain and partial inhibitory activity toward full length MMP-9. Pharmacokinetic evaluation showed that PEGylation extended the plasma half-life of rhTIMP-1 in mice from 1.1 h to 28 h. In biological assays, PEG_20K_-TIMP-1 inhibited both MMP-dependent cancer cell invasion and tumor cell associated gelatinase activity. Overall these results suggest that PEGylated TIMP-1 exhibits improved potential for development as an anti-cancer recombinant protein therapeutic, and additionally may offer potential for clinical applications in the treatment of other diseases.

## Introduction

The **m**atrix **m**etallo**p**roteinases (MMPs) are a family of 23 zinc-dependent endopeptidases with important functions in tissue morphogenesis, wound healing, and other physiological processes that require remodeling of the extracellular matrix [1], [2], [3], [4]. MMP activity is regulated *in vivo* by a family of four endogenous protein protease inhibitors, the **t**issue **i**nhibitors of **m**etallo**p**roteinases (TIMPs), that bind to MMPs in 1∶1 stoichiometry and block the protease active site [5], [6]. Disruption of the balance between MMPs and TIMPs is evidenced under many pathological conditions, and excess MMP activity has long been recognized for important contributions to the development and progression of many diseases including cardiovascular, vascular, and pulmonary diseases, arthritis, multiple sclerosis, and cancer [2], [7], [8], [9], [10].

Diverse roles in disease development and progression have led MMPs to be regarded as promising therapeutic targets, resulting in development of many small-molecule MMP inhibitors, but clinical trials of early-generation MMP inhibitors in cancer and arthritis proved disappointing [11], [12], [13], [14]. Broad-spectrum MMP inhibitors produced serious dose-limiting musculoskeletal toxicity, failed to reach therapeutic plasma levels, and failed to extend progression-free survival in cancer trials [11], [12], [14]; these disappointing outcomes have been attributed both to the toxicity and off-target effects of the drugs and to inadequate specificity for target MMPs.

A less toxic alternative to synthetic MMP inhibitors might be offered by TIMPs. Studies using many preclinical cancer models have shown that overexpression of natural TIMPs in tumors often leads to reduced tumor growth and metastasis [15]. Systemic gene transfer of TIMPs in animal models of cancer has likewise produced antitumor effects, with minimal toxicity [15]. In a handful of studies investigating the suppressive effect of TIMP-1 on tumor cell proliferation and metastasis, mice have been treated with recombinant human TIMP-1 (rhTIMP-1) protein at doses of 2–50 mg/kg with no reported toxicity [16], [17], [18], [19]. Recombinant human TIMPs -1 and -2 have also been investigated as inhibitors of airway inflammation in a murine model of asthma, via intranasal instillation, with promising results [20].

For many applications, one barrier that will likely need to be addressed for TIMPs to enter the clinic as recombinant therapeutics is the short half-life in circulation of these small proteins. Persistence in the circulation is desirable because protein therapeutics generally cannot be administered orally and typically are administered by subcutaneous, intramuscular, or intravenous injection or infusion. Animal studies using recombinant TIMPs have thus far been limited in part by rapid clearance of the protein; the plasma clearance of murine TIMP-1 in rats was reported to occur within minutes [21], and the blood elimination half-life of human TIMP-1 in mice was reported to be ∼4 hours [22].

Chemical modification has been used to improve the pharmacokinetic profiles of several protein therapeutics now in the clinic [23], [24], [25], [26]; one successful strategy is PEGylation, the covalent conjugation of polyethylene glycol chains to a protein. In general, PEGylation reduces renal clearance, increases circulatory half-life by a factor of 5–100-fold, and improves biological activity; it may also confer resistance to proteolysis and reduce immunogenicity [23], [24]. While some PEGylated molecules demonstrate decreased binding in vitro to their natural ligands or receptors, these effects tend to be offset in vivo, with striking improvements in functional pharmacodynamic properties [23], [24]. Furthermore, losses in target affinity can sometimes be minimized by site-directed PEGylation [23]; for example, by chemical conjugation of activated PEG to an unpaired cysteine residue introduced through genetic engineering [27], [28].

TIMP-1 is a potent biological inhibitor of MMPs including MMP-9 (gelatinase B), a metalloproteinase that has been implicated as a potential therapeutic target in a wide variety of inflammatory and vascular diseases and in cancer [29]. Here, we tested several approaches to the covalent PEGylation of rhTIMP-1, and evaluated PEGylated rhTIMP-1 for retention of MMP inhibitory activity in biochemical and biological assays, as well as the impact of PEGylation on circulation half-life in mice.

## Materials and Methods

### Ethics Statement

Animal studies were carried out in strict accordance with the recommendations in the Guide for the Care and Use of Laboratory Animals of the National Institutes of Health. Mice were maintained following approved Mayo Clinic Institutional Animal Care and Use Committee protocols A12409 and A23108. Surgery was performed under avertin anesthesia, acetaminophen was administered throughout the preoperative and postoperative period, and all efforts were made to minimize suffering. For the serial blood withdrawals in the pharmacokinetic study, care was taken not to exceed the guidelines recommended by NIH for non-terminal blood withdrawal from rodents.

### Recombinant Proteins

Mature secreted full-length rhTIMP-1 was expressed using the pTT/TIMP-1 construct transfected into HEK 293E cells [30], and was purified by SP-Sepharose chromatography as we have described previously [31]. Four mutant pTT/TIMP-1 constructs were generated to introduce a free Cys residue (R180C, S181C, Q182C, and A184C) using the Stratagene QuikChange mutagenesis kit (Agilent Technologies, Wilmington, DE, USA) according to the manufacturer’s protocols. TIMP-1-R180C was the most highly expressed mutant in small scale studies; this mutant was produced in large scale for PEGylation studies and purified either by (a) SP-Sepharose chromatography in 50 mM 2-(N-morpholino)ethanesulfonic acid (MES), pH 6.0, using a linear gradient of 0–0.5 M NaCl, or (b) Q-Sepharose chromatography in 20 mM ethanolamine, pH 9.0, using a linear gradient of 0–0.5 M NaCl. Eluted fractions containing the rhTIMP-1 or rhTIMP-1-R180C were assessed by silver stained SDS-PAGE. Recombinant human MMP-3 catalytic domain (MMP-3cd) was expressed in *E. coli*, purified, refolded, and activated as described previously [31], [32]. Full-length recombinant human MMP-9 was purchased from Calbiochem, San Diego, CA, USA.

### Conjugation of PEG to rhTIMP-1-R180C via Cys Residues

The rhTIMP-1-R180C mutant protein (∼11 µM) was incubated in test reactions with 1.5–50-fold molar excess of freshly dissolved monomethoxypolyethylene glycol (mPEG)- maleimide of molecular weight 30K (Laysan Bio Inc, Arab, AL) in 100 mM sodium phosphate at pH 6, pH 7, or pH 8 at room temperature for 15 min –24 h. Prior to some incubations, the rhTIMP-1-R180C protein was pre-treated with the following reducing agents for 10 min –1 hr at room temperature or 37°C with or without inclusion of 2.5 M urea: 1.25–5-fold molar excess of TCEP, 50–200 µM DTT, 0.1–10 mM 2-mercaptoethylamine (MEA), bead-immobilized TCEP (Pierce Biotechnology, Rockford, IL, USA), or bead-immobilized DTT (Cleland’s Reductacryl reagent, Calbiochem). Reducing agents were removed using a mini protein desalting spin column (Pierce) prior to test PEGylations. The degree of PEG incorporation into protein was assessed by observing molecular weight shifts on 10% SDS-polyacrylamide gels; proteins were visualized by silver staining and PEG was visualized by barium iodide staining [33]. Briefly, gels were washed twice with water, incubated with 5% BaCl_2_ for 10 min, and then developed with 0.1 N iodine solution. Retention of MMP inhibitory activity by rhTIMP-1-R180C following partial reduction and/or PEGylation was assessed in assays measuring inhibition of MMP-3cd.

### Conjugation of PEG to rhTIMP-1 via Lys Residues

Activated mPEG-succinimidyl carboxymethyl ester (mPEG-SCM) of molecular weight 5K and 20K (Jenkem Technologies, Allen, TX, USA) were dissolved in dry DMSO to make stock solutions of 40 mM and 10 mM, respectively. Wild-type rhTIMP-1 protein (∼13 µM) was incubated in test reactions with 2–100-fold molar excess of mPEG_5K_-SCM or 1–5-fold molar excess of mPEG_20K_-SCM in 100 mM sodium phosphate at pH 8 or pH 8.5, at room temperature for 30 min –1 h. The degree of PEG incorporation into protein and retention of MMP inhibitory activity were assessed as described above. For larger scale PEGylation reactions, rhTIMP-1, at a concentration of 1 mg/ml quantified by UV absorbance at 280 nm and confirmed by BCA protein assay kit (Pierce), was dialysed into 100 mM sodium phosphate, pH 8.0. The rhTIMP-1 protein was incubated with 5-fold molar excess of mPEG_20K_-SCM or 100-fold molar excess mPEG_5K_-SCM for 30 minutes at room temperature.

To purify PEG_20K_-TIMP-1, the PEGylation reaction was diluted in buffer A (50 mM MES, pH 6.0) to reduce the DMSO concentration to 1.5% and then resolved by SP-Sepharose chromatography. Unreacted rhTIMP-1, PEG_20K_-TIMP-1 and mPEG_20K_-SCM hydrolysis products were separated using a linear gradient of buffer B (50 mM MES, pH 6.0+0.5 M NaCl). A similar approach was used to purify PEG_5K_-TIMP-1 but with both buffers at pH 5.0. Purity and extent of PEGylation was assessed on 10% SDS-polyacrylamide gels silver stained for protein and stained with barium iodide for PEG [33], as described above. Concentrations for unmodified and PEGylated TIMP preparations were determined by UV absorbance at 280 nm using a Nanodrop spectrophotometer (Thermo Fisher Scientific, Wilmington, DE, USA) using the predicted molar extinction coefficient of 26,190 M^−1^cm^−1^ which was calculated using the ExPASy ProtParam tool [34]. Retention of MMP inhibitory activity by PEG_20K_-TIMP-1 and PEG_5K_-TIMP-1 was assessed in assays measuring inhibition of MMP-3cd. PEG_20K_-TIMP-1 was also evaluated for inhibitory activity *versus* full-length recombinant human MMP-9.

### Mass Spectrometry

Distribution of PEGylated species in preparations of PEG_20K_-TIMP-1 and PEG_5K_-TIMP-1 was analyzed on a Bio-Rad ProteinChip SELDI time-of-flight system. Protein samples at 1 mg/ml were mixed in 1∶4 protein-to-matrix volume with the matrix (saturated sinapinic acid in 50% acetonitrile and 0.1% trifluoroacetic acid), and spotted (1 µL) onto a SELDI ProteinChip Gold Array (A-H Format, Bio-Rad). The mass-charge ratios (*m/*z) of TIMP-PEG species were determined using external calibration standards, All-In-One Protein Standards II (Bio-Rad) consisting of recombinant hirudin (7 kDa), cytochrome c (12 kDa), myoglobin (17 kDa), carbonic anhydrase (29 kDa), enolase (47 kDa), albumin (66 kDa) and IgG proteins (147 kDa).

### MMP Inhibition Assays

The activities of PEGylated TIMP-1 species were assessed in MMP inhibition assays monitoring cleavage of the MMP thiopeptolide substrate Ac-Pro-Leu-Gly-S-Leu-Leu-Gly-OEt (Enzo Life Sciences, Plymouth Meeting, PA, USA) as previously described [31]. Briefly, mixtures of MMP-3cd and varying molar ratios of PEGylated or unmodified rhTIMP-1 were preincubated at 37°C for 2h and then mixed with substrate (100 µM) in assay buffer (50 mM HEPES, pH 6.0, 10 mM CaCl_2_, 0.05% Brij-35) containing 1 mM 5,5′-dithiobis(2-nitrobenzoic acid). Linear initial rates were measured continuously as the increase in absorbance at 412 nm on a Varian Cary 100 spectrophotometer (Varian Inc, Palo Alto, CA) or on an Agilent 8453 spectrophotometer (Agilent Technologies, Santa Clara, CA, USA). The final enzyme concentration in the assay was 5 nM. Assays for inhibition of MMP-9 were conducted similarly, except that the assay buffer was at pH 7.0 and the final enzyme concentration in the reaction was 1 nM. The molar equivalents of unmodified or PEGylated rhTIMP-1 required to fully inhibit MMP activities were obtained from slopes fitted using linear regression with Prism 4 (GraphPad Software, San Diego, CA, USA).

### Cell Culture

Human MDA-MB-231-luc2 breast cancer cells (Caliper Life Sciences, Hopkinton, MA, USA) were grown in Eagle’s MEM medium with 10% FBS at 37°C in 5% CO_2_ to 80% confluency [35]. RMF/EG human mammary fibroblasts, derived from a reduction mammoplasty and immortalized with human telomere and GFP [36], were a gift from Charlotte Kuperwasser, Tufts University, Boston, MA, USA. RMF/EG fibroblasts were cultured in DMEM medium containing 10% bovine calf serum and 1% penicillin/streptomycin to 80% confluency.

### Matrigel Transwell Invasion Assays

MDA-MB-231-luc2 cells were split and replated at a density of 2×10^6^ cells per 100 mm dish on the day before the assay. Cells (2.5×10^4^ per well in 500 µL Eagle’s MEM containing 0.1% BSA) were plated into a BD BioCoat Matrigel Invasion Chamber 24 well plate with 8.0 µm PET membrane (BD Falcon, Franklin Lakes, NJ, USA). The lower chambers contained 750 µL/well of NIH/3T3 cell conditioned serum free medium (DMEM supplemented with 50 µg/mL ascorbic acid) as chemo-attractant. Some wells included either rhTIMP-1 or PEG_20K_-TIMP-1 at 50 nM or 500 nM; each condition was represented by 3 replicate wells. Assays were incubated 18 h at 37°C in 5% CO_2_. Non-invading cells were removed from the insert by scrubbing with a cotton swab, and then cells on the lower surface of the filter were fixed with methanol, stained with crystal violet, and counted using Image-Pro 6.3 software (Media Cybernetics) as previously described [37].

### *In Situ* Zymography

*In situ* zymography techniques were modified from previously published protocols [38], [39], [40]. For *in situ* zymography of cultured RMF/EG mammary fibroblasts, 2×10^4^ cells were grown overnight in 500 µL DMEM on 8-well chamber slides (Thermo Scientific, Waltman, MA, USA) at 37°C with 5% CO_2_ under humidified conditions. Incubation solutions containing 50 µg/mL DQ-gelatin (Thermo Scientific) and 1 µg/mL Hoechst 33258 (Invitrogen, Grand Island, NY, USA) were prepared in 1×PBS, with or without 500 nM PEG_20K_-TIMP-1. After removing the media from the cells, 60 µL of incubation solution was added drop-wise to each chamber well to cover all cells. Slides were kept protected from light at room temperature for 1 h, and then examined by fluorescence microscopy and photographed. DQ-gelatin cleavage by gelatinases was visualized using the GFP-channel; GFP expression by the fibroblasts produced a barely detectable background signal, against which the brighter dequenched fluorescein signal from cleaved DQ-gelatin was clearly detected.

For *in situ* zymography of frozen tumor sections, tissue was cryosectioned at −20°C, mounted onto glass slides, and then thawed slowly on ice. An incubation solution containing 100 µg/mL DQ-gelatin and 1 µg/mL Hoechst 33258 in 1×PBS was pre-mixed and equilibrated to 42°C just before mixing with an equal volume of 2% low melting agarose. Incubation solution/agarose (40 µL) was carefully spread over the thawed tissue. Slides were covered from light and placed at 4°C for 5 minutes for the agarose to solidify, followed by 3 hours incubation at room temperature. DQ-gelatin cleavage by gelatinases was assessed via microscopy as described above.

### Pharmacokinetic Study

Mice (6 per group) were injected intraperitoneally with 2 mg/kg rhTIMP-1 or PEG_20K_-TIMP-1. This dose was selected because it is a dose that for rhTIMP-1 has been shown to have an antitumorigenic effect in a xenograft model of colon cancer [19], and in our preliminary studies we confirmed our ability to detect the resultant blood concentration of rhTIMP-1 by ELISA. To minimize the number of mice and quantity of recombinant protein required, blood collections were performed serially using previously described techniques [41], [42], [43]. Mice were placed in a mouse tail illuminator (Braintree Scientific Inc, Braintree, MA, USA) and the tail cleaned with alcohol. After locating a suitable vein, the area was covered with petroleum jelly and a small incision made with a 22½ gauge needle. A 20 µL sample was collected using a heparinized capillary tube and dispensed into a pre-weighed microvette tube precoated with EDTA (Sarstedt, Newton, NC, USA). Within each injection group, mice were divided into two subgroups of 3 (A and B subgroups) which were bled at alternating time points to avoid exceeding recommended volume limits for non-terminal blood withdrawal. For the rhTIMP-1 group mice were bled at 0 min (A and B), 30 min (A), 45 min (B), 1 h (A), 1.5 h (B), 2 h (A), 2.5 h (B), 3 h (A), 4 h (A), 6 h (B), and 8 h (B). For the PEG_20K_-TIMP-1 group mice were bled at 0 min (A and B), 1 h (A), 2 h (B), 3 h (A), 4 h (B), 6 h (A), 8 h (B), 12 h (A), 24 h (A), 48 h (B), and 72 h (B). At the final time point each mouse was terminally bled by cardiac puncture and ∼1 mL of blood was collected.

Blood samples were diluted with 10 volumes 0.1 M trisodium citrate to inhibit coagulation and centrifuged to pellet red blood cells, and then the plasma supernatant was collected and frozen at −30°C until analyzed. Concentrations of TIMP-1 and PEG_20K_-TIMP-1 were quantified using a Human TIMP-1 ELISA kit (Bender MedSystems, San Diego, CA) according to the manufacturer’s instructions, using the manufacturer provided rhTIMP-1 as well as our lab-produced rhTIMP-1 and PEG_20K_-TIMP-1 to generate standard curves. This kit was found to be highly sensitive for both rhTIMP-1 and PEG_20K_-TIMP-1, and to have minimal cross-reactivity with mouse TIMP-1. The dynamic range for detection of rhTIMP-1 and PEG_20K_-TIMP-1 was 39–2500 pg/mL; these concentrations were achieved in plasma samples after an additional 50–500-fold dilution in the assay (after the initial 10-fold dilution). Standard curves were fit to data for 8 rhTIMP-1 concentrations run in duplicate, using a four-parameter sigmoidal (logistic) model (R^2^>0.99), and concentrations in plasma were determined from duplicate assays by nonlinear interpolation from the standard curve, using Prism version 4.0 (GraphPad Software, La Jolla, CA, USA). The data were displayed as a semilogarithmic plot and fitted to the equation for two phase exponential decay using Prism version 4.0 to obtain the best fit values for distribution and elimination; values were excluded for rhTIMP-1 for the 30 min and 45 min time points, where the measured values exceeded the quantifiable range of the assay.

### Mammary Orthotopic Xenograft Study

A mixture of 1×10^5^ MDA-MB-231-luc2 human breast cancer cells and 1×10^5^ human mammary fibroblasts in 25 µL serum-free Eagle’s MEM medium plus 25 µL growth factor-reduced phenol red-free Matrigel was injected into the inguinal mammary gland of 6–10-week-old female Nod/LtSz-prkds(scid) (NOD/SCID) mice (Jackson Laboratory, Bar Harbor, ME, USA). Tumors were allowed to grow over the course of 11 weeks, monitored weekly by bioluminescence imaging using an IVIS Spectrum 3-dimensional imaging system (Caliper Life Sciences).

PEG_20K_-TIMP-1 was dialyzed into PBS, filter sterilized, and protein concentration was determined by UV absorbance at 280 nm using a Nanodrop spectrophotometer (Thermo Fisher Scientific, Wilmington, DE, USA). At 11 weeks post-implantation, two tumor-bearing mice were injected intraperitoneally with 2 mg/kg PEG_20K_-TIMP-1 and two tumor-bearing mice were injected with 0.9% saline only. This dose was selected because it is a dose that for rhTIMP-1 has been shown to have an antitumorigenic effect in a xenograft model of colon cancer [19]. Twenty four hours later, all four mice were euthanized by CO_2_ inhalation, and tumors were resected and flash frozen in liquid N_2_ and stored at −80°C until analysis. MMP activity was assessed in fresh-frozen tumors by *in situ* zymography as described above.

## Results

**Figure 1:**
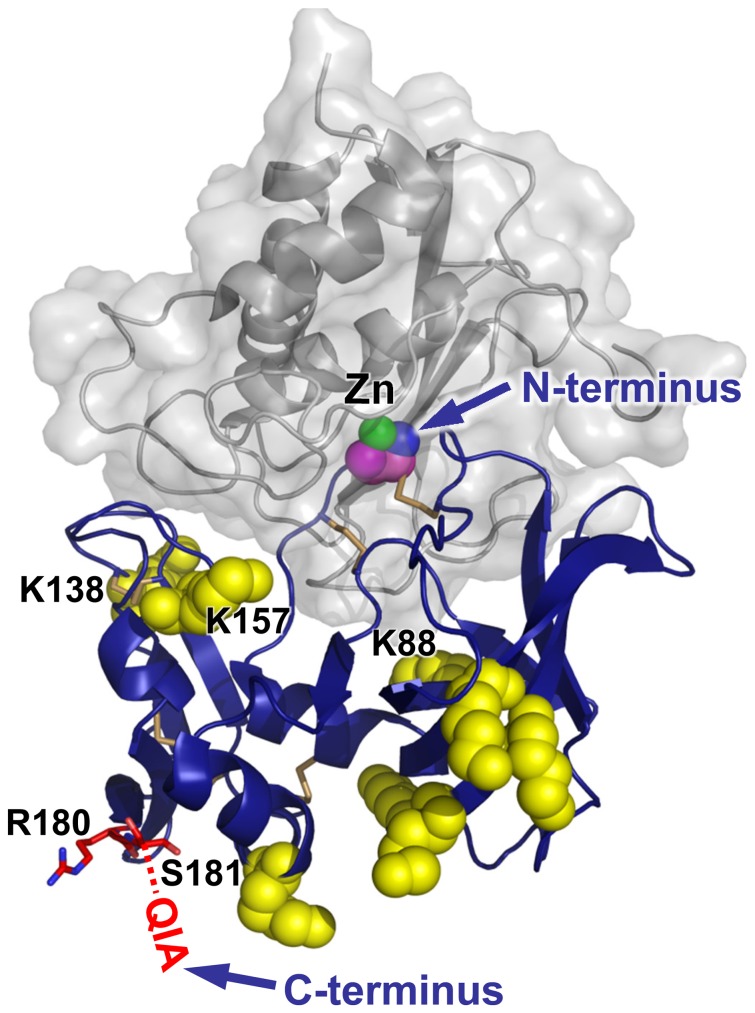
TIMP-1 sites of inhibitory interaction and PEGylation. TIMP-1 (blue) is shown binding to the MMP-3 catalytic domain (gray); the N-terminal Cys-1 backbone (magenta and blue spheres) coordinates directly to the MMP catalytic Zn (green). The C-terminal residues Arg-180, Ser-181, Gln-182, and Ala-184 (red sticks; residues 182–184 unstructured in the crystal structure) were initially mutated to Cys and targeted for site-specific PEGylation. In a subsequent approach, the 8 natural Lys residues (yellow spheres) were targeted for PEGylation. Lys-138, Lys-157 and Lys-88 all lie within 10 Å of the MMP-3 cd, while other Lys residues are distant from the binding interface. Structure coordinates are from PDB ID 1UEA [44]; figure was created using PyMOL.

In considering how best to approach PEGylation of rhTIMP-1, we analyzed the structure of the protein and its mode of interaction with an MMP catalytic domain (**Fig. 1**) [31], [44]. TIMP-1 is a globular protein of 184 amino acids and is glycosylated on two Asn residues. It possesses 12 Cys residues, paired as 6 disulfide bonds, and 8 Lys residues. The most common approach to protein PEGylation involves coupling of activated PEGs to primary amines found on Lys side chains and at the protein N-terminus [45]. In the case of TIMP-1, the N-terminal amine is absolutely essential for activity, because it inserts into the active site cleft of the protease and directly coordinates to the catalytic zinc (**Fig. 1**); this interaction is the basis for inhibition of MMPs by TIMPs. Any modification of the natural TIMP N-terminus, even extension by a single Ala residue, will destroy antiprotease activity [46], and mutations that disrupt the N-terminal disulfide bond between Cys-1 and Cys-70 are similarly destructive [47], [48]. To avoid the risk of N-terminal PEGylation, and to direct PEG attachment to a site distant from the TIMP-MMP interface, our first approach was to introduce a single unpaired Cys residue near the flexible C-terminus of TIMP-1, opposite the MMP-binding face, for reaction with a thiol-reactive PEG.

### Conjugation of PEG to rhTIMP-1 via an Introduced Cys Residue

To facilitate site-specific PEGylation of rhTIMP-1, we made four mutants each introducing a Cys residue near the C-terminus (**Fig. 1**) and tested expression levels in the HEK 293E mammalian expression system, finding highest expression for the R180C mutant (**Fig. S1A**). The rhTIMP-1-R180C was produced in larger scale, purified, and analyzed under nonreducing conditions, which showed that while some disulfide dimers were present, the major species was monomeric (**Fig. S1B**), with the introduced Cys presumed to be available for modification. An mPEG-maleimide reagent of molecular weight 30K was selected with the rationale that attachment of a single very large PEG substituent would likely be sufficient to prevent rapid renal elimination.

Reaction conditions were tested using a variety of PEG:protein molar ratios, pH ranges, temperatures, and reaction times. Surprisingly, we failed to detect rhTIMP-1-R180C PEGylation under any conditions. We verified 100% reactivity of the purchased mPEG-maleimide by titration against cysteine using Ellman’s reagent (5,5′-dithiobis-(2-nitrobenzoic acid) to quantify residual free cysteine. We next hypothesized that failure of rhTIMP-1-R180C to react could be attributable either to (a) inaccessibility of Cys-180 caused by the fold of the protein, or (b) covalent blockage of Cys-180 by disulfide formation with small sulfhydryl compounds. To distinguish between these possibilities, we carried out test reactions in the presence of urea to partially denature the protein, alleviating steric hindrance, or TCEP, a disulfide reductant unreactive with mPEG-maleimide. Inclusion of TCEP resulted in complete PEGylation while urea denaturation had little effect, suggesting that the difficulties in PEGylating rhTIMP-1-R180C were caused solely by Cys oxidation.

In an attempt to identify regioselective conditions for partial reduction that would allow deprotection of Cys-180 without disrupting the native disulphide bonds essential for TIMP-1 activity [47], [48], we tested a wide variety of reducing agents and conditions, assessing both the effectiveness of PEGylation and the retention of MMP inhibitory activity following reduction. As summarized in **Table S1**, regardless of the reducing agent employed, conditions resulting in efficient PEGylation of rhTIMP-1-R180C also severely compromised MMP inhibitory activity. We concluded that PEGylation on Cys-180 of the mutant rhTIMP-1 was incompatible with retention of activity.

### Conjugation of PEG to rhTIMP-1 via Native Lys Residues

As an alternative approach for PEGylation of rhTIMP-1, we attempted to identify conditions under which Lys residues on the surface of the protein could be modified with minimal disruption of activity. Because the free N-terminal amine is vital for the inhibitory function of TIMP-1, conditions were optimized to favor modification on Lys residues and to avoid modification of the N-terminus. There are 8 Lys residues in TIMP-1 which could potentially serve as sites of modification (**Fig. 1**). The majority are located distant from the MMP interface where modification would not be expected to interfere with activity; however, Lys-88, Lys-138, and Lys-157, with respective distances of 8Å, 5Å, and 6Å from a bound MMP catalytic domain, could conceivably hinder MMP binding upon PEGylation. We therefore tested the impact on rhTIMP-1 activity of modification by two molecular weights of activated amine reactive mPEG (mPEG-SCM-5K and mPEG-SCM-20K) under conditions allowing varying degrees of PEGylation.

**Figure 2:**
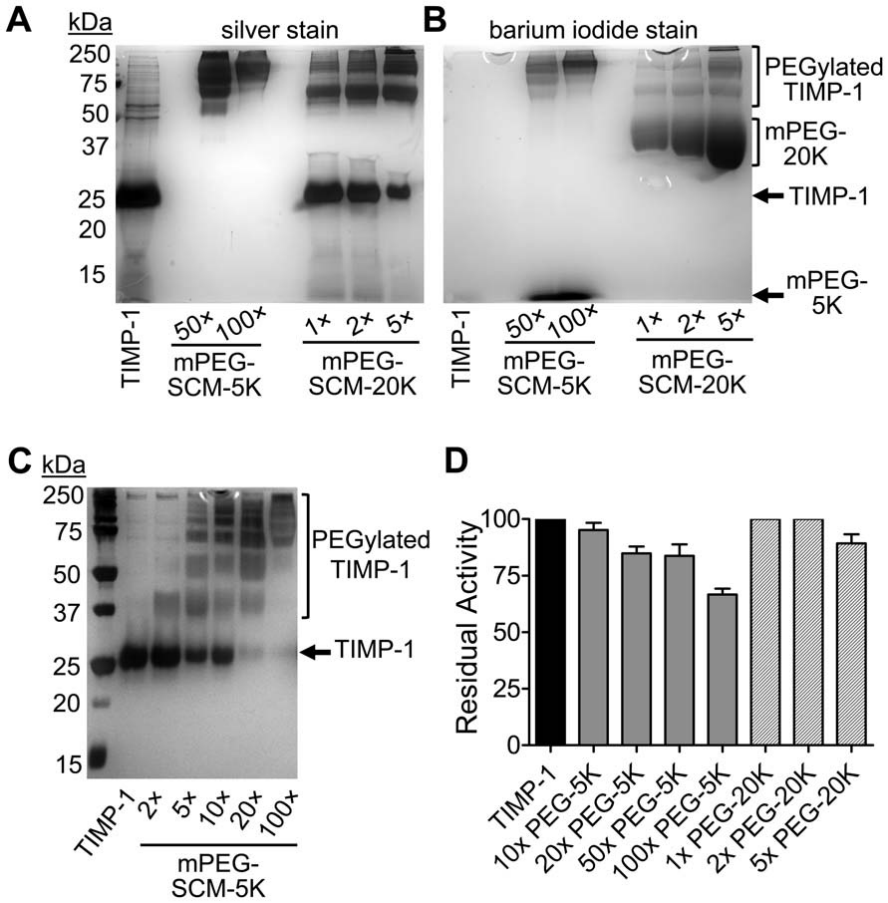
PEGylation on rhTIMP-1 Lys residues with mPEG-SCM reagents. (A) Silver stained gel shows unmodified rhTIMP-1 and PEGylation reactions carried out in the presence of 50–100× molar excess of mPEG-SCM-5K or 1–5× molar excess of mPEG-SCM-20K, as indicated beneath gel. (B) The same gel stained with barium iodide shows the electrophoretic migration of mPEG-5K and mPEG-20K hydrolysis products and PEGylated rhTIMP-1 species. (C) Silver stained gel shows a concentration-dependent increase in molecular weight upon PEGylation with mPEG-5K-SCM in increasing molar excess. (D) Graph shows retention of MMP-3cd inhibitory activity relative to rhTIMP-1 by PEGylated rhTIMP-1 species from the reactions shown in panels A-C. The molar excess over rhTIMP-1 and molecular weight of the activated PEG in each reaction is indicated below the gel.

We observed a concentration-dependent increase in PEGylation (both fraction of protein PEGylated and molecular weight distribution the of PEGylated species) with increasing molar excess of reagents mPEG-SCM-5K or mPEG-SCM-20K (**Fig. 2A–C**). Reactions were assessed by SDS-PAGE, evaluating by silver staining the change in apparent molecular weight of rhTIMP-1 protein upon modification (**Fig. 2A,C**) and confirming by barium iodide staining the incorporation of PEG into the higher molecular weight protein species (**Fig. 2B**). In reactions with mPEG-SCM-5K, essentially all rhTIMP-1 became PEGylated in the presence of 20×or greater molar excess of reagent (**Fig. 2A,C**). Activity tests showed retention of >80% MMP inhibitory activity in PEGylation with 20×or 50×excess reagent and 67% activity with 100×excess reagent (**Fig. 2D**). For mPEG-SCM-20K, the greatest molar excess of reagent achieved was 5×, yielding a mixture of mono- and di-PEGylated species with 10–20% residual unreacted rhTIMP-1 (**Fig. 2A**); this mixture retained 90% of the original activity (**Fig. 2D**).

### Production and Characterization of PEG_5K_-TIMP-1 and PEG_20K_-TIMP-1

**Figure 3:**
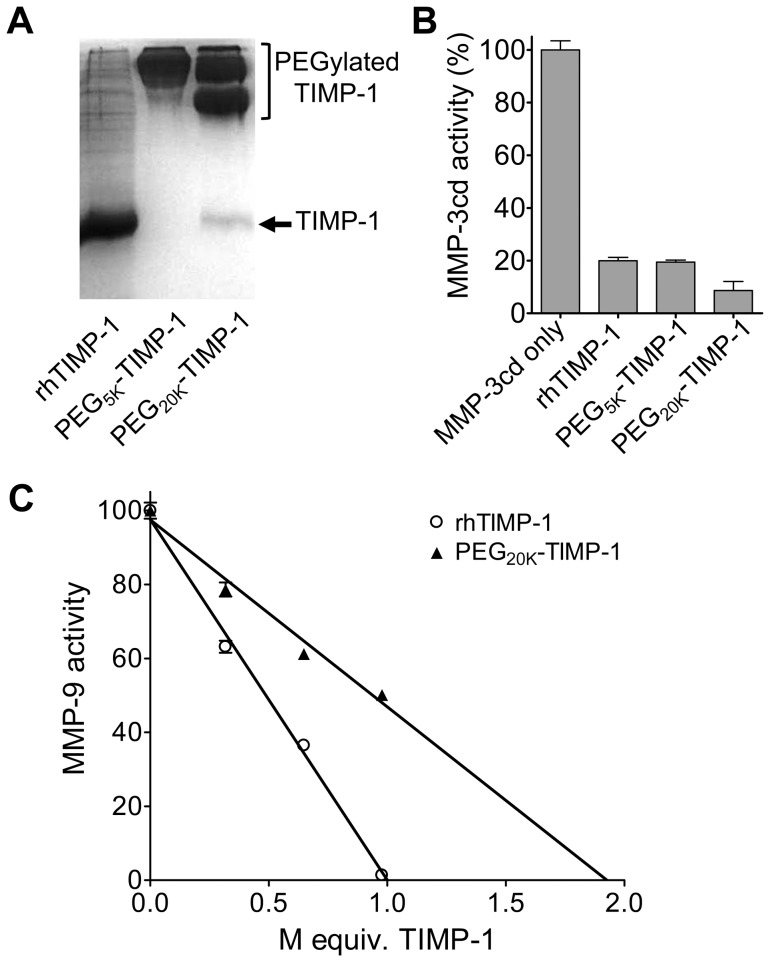
MMP inhibitory activity of PEGylated rhTIMP-1. (A) Silver stained gel shows purified rhTIMP-1, PEG_5K_ -TIMP-1, and PEG_20K_ -TIMP-1. Reactions were carried out using 100× molar excess of mPEG-SCM-5K or 5× molar excess of mPEG-SCM-20K, and PEGylated protein species were purified by ion exchange chromatography. (B) Graph shows inhibition of MMP-3cd by 0.8 molar equivalent of rhTIMP-1, PEG_5K_ -TIMP-1 or PEG_20K_ -TIMP-1. Protein concentrations were calculated from absorbance measurements using predicted molar extinction coefficients. (C) Graph shows titration of a fixed concentration of full-length recombinant human MMP-9 by increasing molar equivalents of rhTIMP-1 and PEG_20K_-TIMP-1.

Larger scale PEGylation reactions were carried out using 100×molar excess of mPEG-SCM-5K or 5×molar excess of mPEG-SCM-20K, and PEGylated proteins were purified by ion exchange chromatography. Preparations were assessed by SDS-PAGE, where the purified PEG_20K_-TIMP-1 revealed a very slight degree of contamination with unmodified rhTIMP-1 (**Fig. 3A**). In activity assays testing the inhibitory activity of the PEGylated TIMP preparations against the MMP-3 catalytic domain (MMP-3cd) when incubated in a 0.8∶1.0 molar ratio, PEG_5K_-TIMP-1 inhibited MMP activity by 80%, identical to unmodified rhTIMP-1 and consistent with 1∶1 stoichiometry of inhibition, indicating full retention of inhibitory activity (**Fig. 3B**). Surprisingly, the PEG_20K_-TIMP-1 preparation inhibited MMP activity to an even greater extent, revealing activity greater than that calculated for 100% activity (**Fig. 3B**). Since it does not seem plausible that the 1∶1 stoichiometry of inhibition will have changed upon PEGylation, we conclude that the discrepancy is likely due to an underestimate of protein concentration in this PEGylated preparation as determined by absorbance measurements at 280 nm. PEGylation does not alter the calculated molar extinction coefficient, but it is possible that the presence of PEG chains at specific sites on rhTIMP-1 alter the chromophore microenvironment of one or more specific aromatic residues, resulting in small changes to the measured extinction coefficient of the natively folded protein.

We also compared PEG_20K_-TIMP-1 to rhTIMP-1 for inhibition of full-length recombinant human MMP-9. We found that whereas 1.0 molar equivalent of rhTIMP-1 effectively quenches the activity of MMP-9, 1.9 molar equivalents of PEG_20K_-TIMP-1 would be required to achieve the same effect (**Fig. 3C**). This suggests that the PEG_20K_-TIMP-1 preparation represents a heterogenous mixture of PEGylated species, in which nearly half of the molecules are PEGylated at a site deleterious for MMP-9 binding. In addition to binding the catalytic domain of MMP-9, TIMP-1 binds to the C-terminal hemopexin domain of MMP-9 and proMMP-9. Although the precise structural nature of this contact is not known, it appears to involve the C-terminal sub-domain of TIMP-1 [49], [50]. Our data suggest that, independently of its effect on MMP catalytic domain binding, TIMP-1 PEGylation might interfere with this secondary interaction and thus alter the specific ability of TIMP-1 to inhibit intact MMP-9.

**Figure 4:**
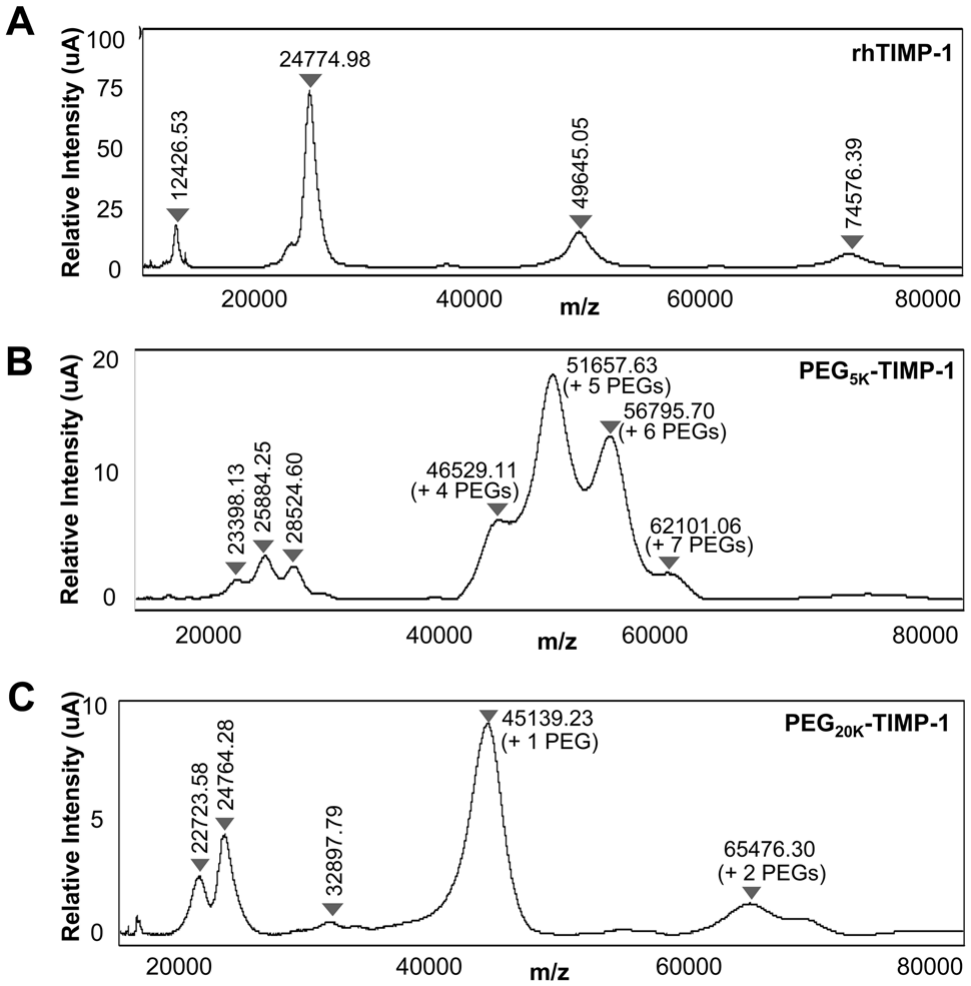
SELDI MS for rhTIMP-1 and PEGylated preparations. (A) SELDI time-of-flight mass spectrum of unmodified rhTIMP-1 shows a molecular mass of 24,775 Da. (B) Mass spectrum of PEG_5K_ -TIMP-1 shows a molecular mass range of 46,529 Da –62,101 Da, indicating conjugation of 4–7 mPEG-5K chains; the most abundant species possesses 5 mPEG-5K chains. The smaller peaks in the range of 23,398–28,524 are consistent with doubly charged ions of the PEGylated species bearing 4–6 mPEG chains. (C) Mass spectrum of PEG_20K_-TIMP-1 shows a molecular mass range of 45,139 Da –65,476 Da, indicating attachment of 1–2 mPEG-20K chains, with the mono-PEGylated species predominant. Doubly charged ions for the mono- and di-PEGylated species are also present, as is a peak for unmodified rhTIMP-1.

Next, we analyzed the distribution of PEGylated species in the PEG_5K_-TIMP-1 and PEG_20K_-TIMP-1 preparations by surface enhanced laser desorption/ionization (SELDI) time-of-flight mass spectrometry. The mass peak for rhTIMP-1 was centered at 24,775 Da (**Fig. 4A**), consistent with the calculated protein mass of 20,709 Da and the two confirmed sites of N-linked glycosylation [51]. For PEG_5K_-TIMP-1, peaks were centered at 46529 Da, 51657 Da, 567951 Da and 62101 Da, confirming the conjugation of 4–7 molecules, respectively, of mPEG-SCM-5K; the most abundant species was that featuring attachment of 5 PEG chains (**Fig. 4B**). For PEG_20K_-TIMP-1, peaks were centered at 45139 Da and 65476 Da, confirming the conjugation of 1–2 molecules of mPEG-SCM-20K, with a predominance of mono-PEGylation; a peak for the residual unmodified rhTIMP-1 was present as well (**Fig. 4C**).

### PEGylation Extends rhTIMP-1 Plasma Half-life in Mice

**Figure 5:**
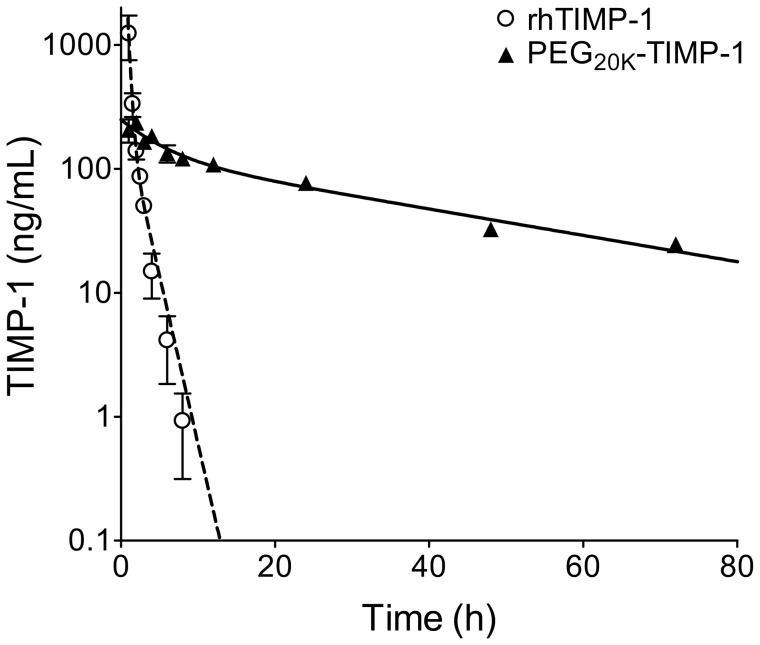
PEG_20K_-TIMP-1 *versus* rhTIMP-1 plasma half-life. Semilogarithmic plot of rhTIMP-1 (open circles) or PEG_20K_-TIMP-1 (filled triangles) in plasma *versus* time show that half-life is markedly extended for the PEGylated protein. Mice (6 per group) were injected intraperitoneally with 2 mg/kg rhTIMP-1 or PEG_20K_-TIMP-1 and then blood samples were collected serially at the indicated time points; each data point represents the average and standard error for measurements from 3 mice. The dotted and solid curves show best fits to the equation for two phase exponential decay for rhTIMP-1 and PEG_20K_-TIMP-1, respectively.

We carried out a pharmacokinetic study in mice to compare the circulation half-life of unmodified and PEGylated rhTIMP-1, which were quantified in plasma using an ELISA assay specific for human TIMP-1. In preliminary studies we found that PEG_20K_-TIMP-1 was detected with similar sensitivity to rhTIMP-1, whereas the more extensively modified PEG_5K_-TIMP-1 was detected with about 5-fold lower sensitivity; we therefore chose to focus on PEG_20K_-TIMP-1 for the *in vivo* studies. Using an experimental design involving serial blood sampling of 6 mice per group, we compared the persistence in circulation of rhTIMP-1 *versus* PEG_20K_-TIMP-1 after a single 2 mg/kg intraperitoneal injection (**Fig. 5**). For each series, data points were fitted by a two phase exponential decay model, yielding for rhTIMP-1 a distribution half-life of 0.22 h and an elimination half-life of 1.1 h; for PEG_20K_-TIMP-1 the distribution half-life was 3.4 h and the elimination half-life was 28 h. These data indicate a 25-fold increase in terminal elimination half-life for the PEGylated TIMP-1. No toxicity or adverse effects were noted in the treated mice.

### PEG_20K_-TIMP-1 Inhibits MMP-dependent Cancer Cell Invasion and Tumor Cell-associated Gelatinase Activity

**Figure 6:**
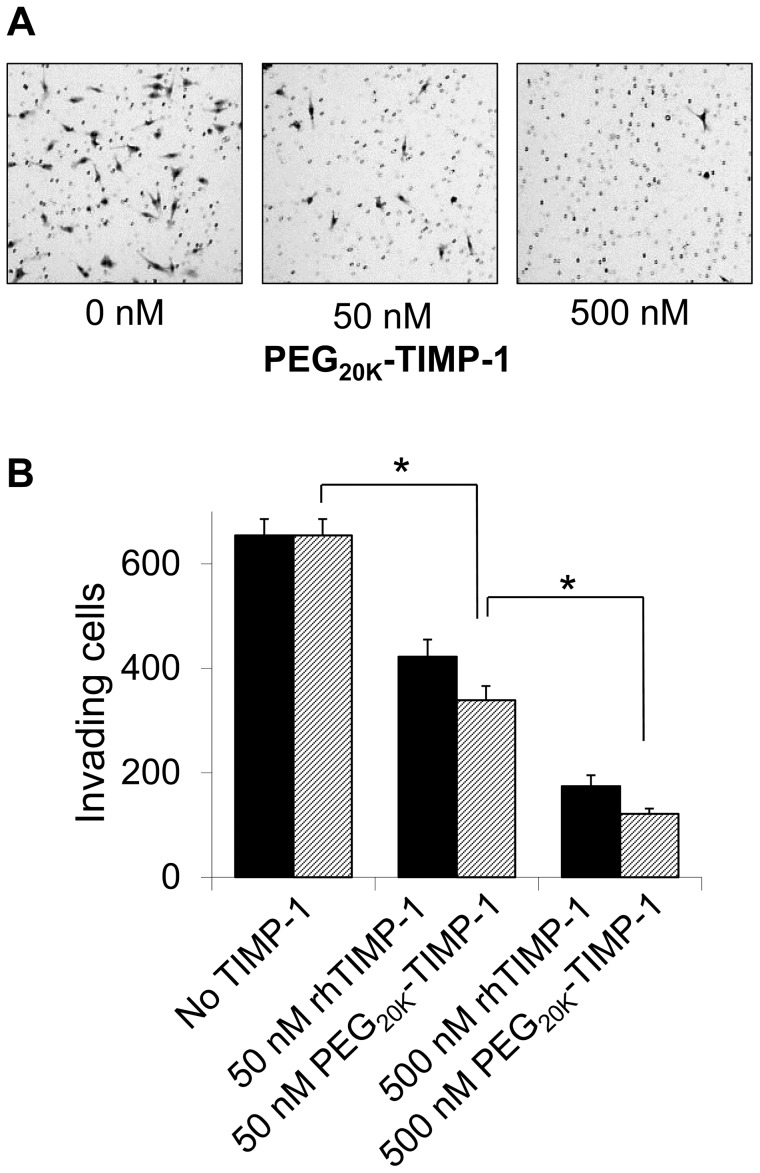
PEG_20K_-TIMP-1 activity in cancer cell invasion assay. (A) Pictures of representative fields from filters with fixed and stained cells are shown for control and PEG_20K_-TIMP-1-treated wells. (B) Graph shows a concentration dependent decrease in the number of invasive MDA-MB-231 cells in the presence of 50 or 500 nM of unmodified or PEGylated rhTIMP-1 in Matrigel transwell assays. Plotted values represent average and standard error from triplicate filters; cells were counted from entire filters using image processing software. *, p<0.01.

The ability of PEG_20K_-TIMP-1 to inhibit MMP-dependent cancer cell invasion was evaluated in Matrigel transwell assays using MDA-MB-231 cells, a highly invasive cell line derived from a metastatic breast adenocarcinoma. The invasive behavior of these cells has been found to be highly dependent on MMPs [52]. Invasion was significantly decreased in a concentration dependent fashion when assays were carried out in the presence of 50 or 500 nM unmodified or PEGylated rhTIMP-1 (**Fig. 6**). Surprisingly, PEG_20K_-TIMP-1 was more effective than rhTIMP-1 for suppression of invasion (**Fig. 6**), despite the reported importance of MMP-9 for invasion in this model [53], [54], [55] and our data showing that MMP-9 inhibition is diminished by PEGylation. These results suggest the possibility that PEGylation may render TIMP-1 more stable against degradation in the cell culture environment.

**Figure 7:**
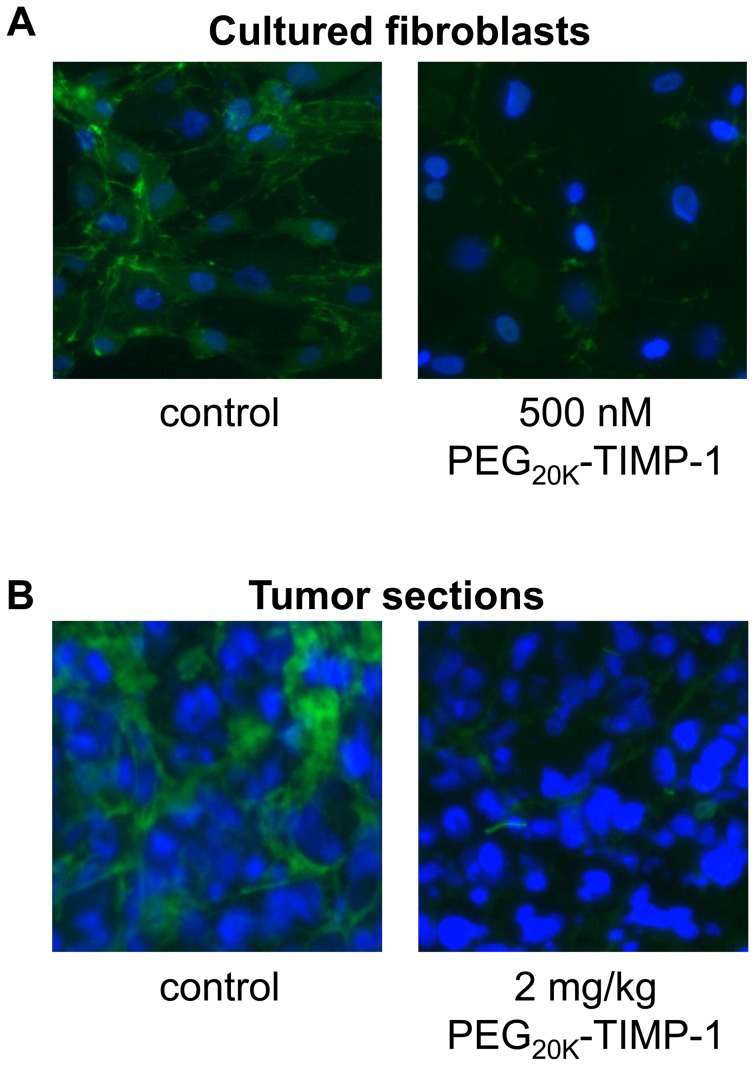
MMP inhibitory activity of PEG_20K_-TIMP-1 in fibroblast cultures and orthotopic tumors. (A) *In situ* zymography of mammary fibroblast cultures developed with (right panel) or without (left panel) 500 nM PEG_20K_-TIMP-1 show decreased gelatinase activity in the treated cells, as indicated by a reduction in green fluorescent signal produced by cleavage of quenched fluorescent substrate DQ-gelatin. (B) *In situ* zymography of tumor sections from mammary tumor-bearing mice injected 24 hours prior to sacrifice with saline (left) or 2 mg/kg PEG_20K_-TIMP-1 (right) show reduced gelatinase activity in the tumor from the mouse receiving PEG_20K_-TIMP-1.

To directly visualize the inhibition of MMP activity in the cellular environment, we carried out *in situ* zymography experiments with human mammary fibroblasts, which secrete high levels of MMP-2 and moderate levels of MMP-9. Using this technique, pericellular gelatinase activity is imaged through cleavage of the quenched fluorogenic substrate DQ-gelatin. While control cultures showed clear evidence of gelatinase activity as indicated by green fluorescence surrounding the cells (**Fig. 7A**, left), greatly reduced gelatinase activity was evidenced in cultures treated with 500 nM PEG_20K_-TIMP-1 (**Fig. 7B**, right).

The ability of the PEG_20K_-TIMP-1 to inhibit tumor-associated MMP activity was also evaluated in mice bearing orthotopic xenograft mammary tumors. Because complex interactions between tumor cells and stromal fibroblasts stimulate MMP production at the invasive front in both types of cells [39], [56], [57], [58], [59], [60], we chose to employ a physiologically relevant model in which human MDA-MB-231 tumor cells and human mammary fibroblasts were co-implanted into the mammary fat pad of immunocompromised mice. Tumors were allowed to grow for 11 weeks, mice bearing similarly sized tumors were then injected with a single dose of either 2 mg/kg PEG_20K_-TIMP-1 or saline only, and MMP activity was assessed by *in situ* zymography in the fresh-frozen tumors harvested 24 h later. Because of the very short elimination half-life observed for rhTIMP-1 in the pharmacokinetic study (**Fig. 5**), we did not include rhTIMP-1 as a control in the tumor targeting study, anticipating that it would show little effect after 24 h *in vivo*. *In situ* zymography of tumors from control mice injected with saline showed gelatinase activity that was most concentrated near the periphery of the tumors (**Fig. 7B**, left), while tumors from PEG_20K_-TIMP-1-injected mice showed noticeably diminished evidence of gelatinase activity associated with the tumor (**Fig. 7B**, right). This experiment suggests that injected PEG_20K_-TIMP-1 is localized to tumors, where it persists at least 24 hours after injection and effectively suppresses gelatinase activity *in vivo* in tumor tissue.

## Discussion

MMPs remain therapeutic targets of interest for cancer and for many other diseases. Recombinant TIMPs represent an as yet underexplored source of biologics that could be developed for clinical uses targeting MMPs. Therapeutics derived from human proteins offer a number of advantages over small-molecule drugs, including greater specificity and low toxicity [61], however they often come with a unique set of challenges with regard to formulation, delivery, *in vivo* stability, short circulation half-life, and rapid clearance [23]. Here, we pursued PEGylation as an approach to overcome the short plasma half-life of rhTIMP-1 and developed methodology for limited PEGylation on Lys side chains of rhTIMP-1 with preservation of MMP inhibitory activity. We found that the resultant PEG_20K_-TIMP-1 preparation inhibited MMP activity *in vitro* and *in vivo*, and was capable of inhibiting cancer cell invasion with improved potency.

Previous reports of unmodified rhTIMP-1 pharmacokinetics in rodents have varied considerably; an early study found an elimination half-life of 4 h in mice [22], while another group recently reported a half-life of 42 h in an ischemia-reperfusion model in rats [62]. Both of these values are considerably longer than the 1.1 h elimination half-life that we measured for rhTIMP-1 (**Fig. 5**). Major differences include (a) that both prior studies employed ^125^I-labelled rhTIMP-1 to follow distribution and clearance while we used an ELISA with high specificity for human TIMP-1, and (b) that the prior studies administered much lower doses (µg/kg rather than mg/kg). A caveat in the interpretation of radiolabelling studies is that the assay does not specifically monitor intact or active rhTIMP-1 molecules, but would detect partially degraded polypeptide fragments of rhTIMP-1 as well. Indeed, electrophoretic analysis of ^125^I-labelled rhTIMP-1 in the rat ischemia-reperfusion model showed that the signal for intact protein peaked at 1.5 h and was much diminished by 3 h [62]. By contrast, we would anticipate that the ELISA used in our experiment would provide a more specific readout for intact biologically active TIMP-1, and therefore a more meaningful measurement from the perspective of therapeutic utility. Our pharmacokinetic study showed gradual elimination of PEG_20K_-TIMP-1 over the course of several days (**Fig. 5**); this is consistent with our *in situ* zymography results (**Fig. 7B**), which suggest that substantial inhibitory activity is retained *in vivo* even after 24 h.

TIMP-1 and its modified derivatives may be particularly well suited for therapeutic targeting of MMP-9, a potential drug target in many pathological processes. MMP-9 has been implicated in atherosclerotic plaque rupture, tissue damage after acute myocardial infarction, and breakdown of the blood-brain barrier and development of brain edema after cerebral ischemia and in other CNS conditions [29]. It also plays significant roles promoting cancer invasion, metastasis, and angiogenesis, as well as inflammatory diseases including osteoarthritis, rheumatoid arthritis, multiple sclerosis, chronic obstructive pulmonary disease (COPD) and other conditions of pulmonary inflammation and fibrosis [29], [63], [64]. Among TIMPs, native TIMP-1 has the strongest preexisting affinity for MMP-9 [50], [65], [66], and possesses the unique ability to bind to proMMP-9 through an interaction between the C-terminal domain of TIMP-1 and the C-terminal hemopexin (PEX) domain of the proenzyme [49], [50]. This high-affinity interaction substantially accelerates the kinetics of MMP-9/TIMP-1 association [50], likely contributing to selectivity of TIMP-1 toward MMP-9 *in vivo*. The N-terminal domain of TIMP-1 in the proMMP-9/TIMP-1 complex remains available and competent for inhibiting other active MMP molecules including MMP-3 [67], [68], a physiological activator of MMP-9 [69], [70]; the interaction with TIMP-1 therefore protects proMMP-9 from enzymatic activation *in vitro* [67], [71] and *in vivo* [72]. While native TIMP-1 therefore offers special advantages where targeting of MMP-9 is desired, we found that the PEG_20K_-TIMP-1 preparation inhibited MMP-9 somewhat less effectively than it inhibited MMP-3cd. This was possibly due to steric incompatibility between one or more sites of TIMP-1 lysine PEGylation and the MMP-9 PEX domain. For retention of optimal activity toward MMP-9, it may be advantageous for future studies to further pursue approaches for more regioselective PEGylation of rhTIMP-1. Although we did not find success with PEGylation of an introduced Cys residue, the most common approach toward site-specific PEGylation, another possible approach might pursue PEGylation on the two glycosyl groups of TIMP-1, both of which lie within the N-terminal domain and well removed from the inhibitory site. Yet another possibility could involve mutational studies to identify which of the TIMP-1 Lys residues interferes with MMP-9 association upon PEGylation, and then specifically removing that site of modification by mutagenesis.

TIMP-1 offers a potential biopharmaceutical MMP inhibitor but is rapidly eliminated; our results indicate that PEGylation is one feasible approach to improve its pharmacokinetic profile while preserving activity. Several recent publications have suggested other biopharmaceutical approaches to MMP inhibition. Nanoparticles loaded with mouse TIMP-1 were shown to provide neuroprotection in an organotypic hippocampal slice culture model [73]. A fusion protein formed from human TIMP-2 and human serum albumin was found to provide an improved pharmacokinetic profile and biodistribution in a tumor model and to provide an antiangiogenic effect [74]. Furthermore, TIMPs are not the only biomolecules to be investigated as scaffolds for development of MMP inhibitors. An inhibitory human antibody targeting MMP-14, developed using phage display technology, has shown *in vivo* activity in mouse xenograft tumor models [75]. Mouse monoclonal antibodies, raised against a synthetic antigen that mimics the MMP catalytic site, were shown to inhibit gelatinases via a TIMP-like binding mechanism, and to show therapeutic promise in a mouse model of inflammatory bowel disease [76]. The methods tested and developed in the present work will contribute to the developing biotechnological arsenal for creating next-generation MMP inhibitors.

## Supporting Information

Figure S1 **Expression and characterization of rhTIMP-1 Cys Mutant R180C.** (a) Silver stained gel shows varying levels of recombinant TIMP in conditioned media from expression cultures for different rhTIMP-1 Cys mutants, as indicated above the gel. (b) Non-reducing SDS-PAGE and Western blot of purified rhTIMP-1-R180C show that the predominant species present is monomeric.
(TIF)

Table S1 Effect of partially reducing conditions on MMP inhibitory activity and PEGylation.
(DOCX)
