# Supplementary figures and images for: PEGylation Extends Circulation Half-Life While Preserving In Vitro and In Vivo Activity of Tissue Inhibitor of Metalloproteinases-1 (TIMP-1)
Source: PLoS One. 2012 Nov 20;7(11):e50028. doi: 10.1371/journal.pone.0050028 (PMC3502186; doi:10.1371/journal.pone.0050028)

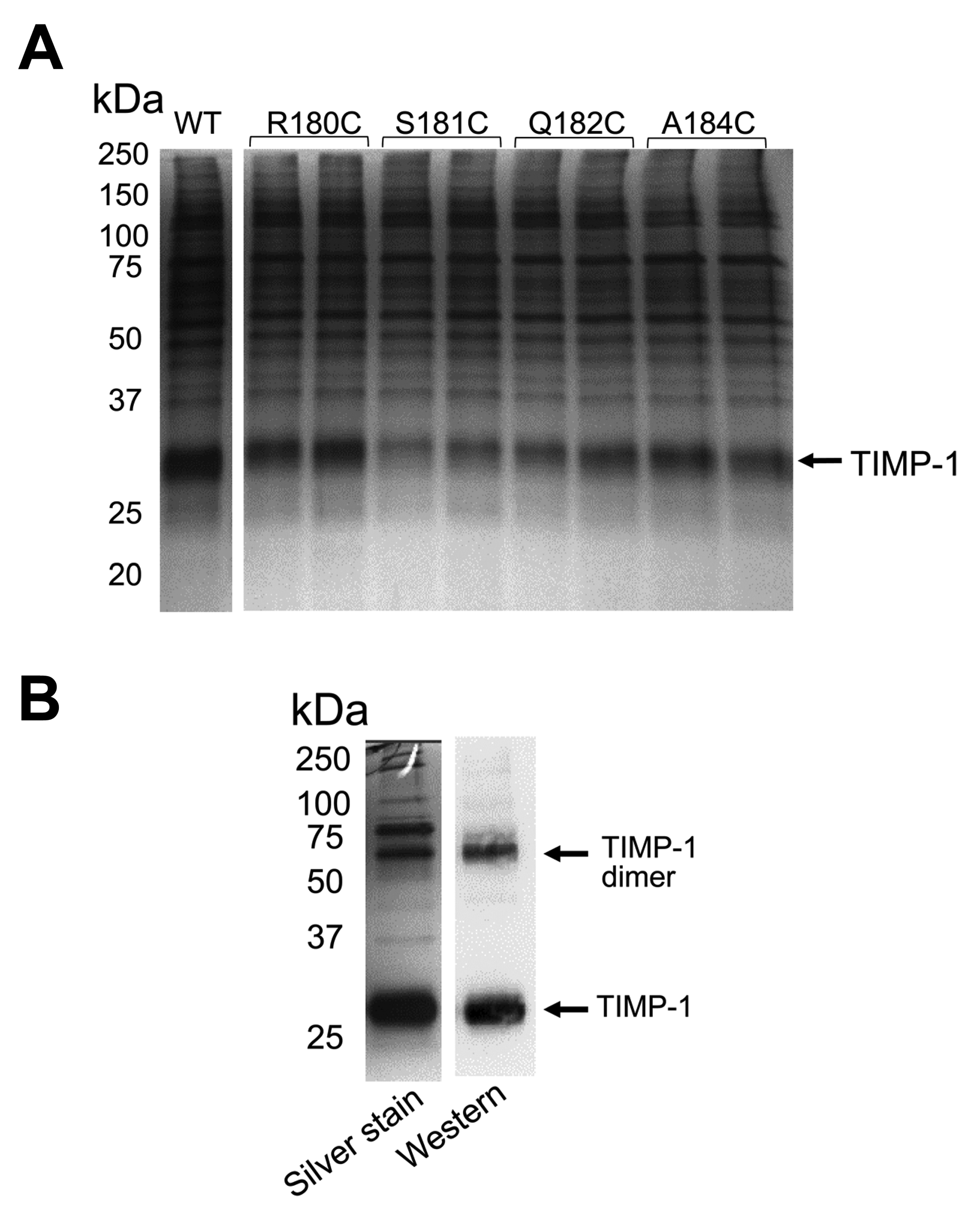

Supplement: Figure S1 — Expression and characterization of rhTIMP-1 Cys Mutant R180C. (a) Silver stained gel shows varying levels of recombinant TIMP in conditioned media from expression cultures for different rhTIMP-1 Cys mutants, as indicated above the gel. (b) Non-reducing SDS-PAGE and Western blot of purified rhTIMP-1-R180C show that the predominant species present is monomeric. (TIF) [file pone.0050028.s001.tif]
