# Supplementary material for: PEGylation Extends Circulation Half-Life While Preserving In Vitro and In Vivo Activity of Tissue Inhibitor of Metalloproteinases-1 (TIMP-1)
Source: PLoS One. 2012 Nov 20;7(11):e50028. doi: 10.1371/journal.pone.0050028 (PMC3502186; doi:10.1371/journal.pone.0050028)
Supplement: Table S1 — Effect of partially reducing conditions on MMP inhibitory activity and PEGylation. (DOCX) [file pone.0050028.s002.docx]

**Table S1**. Effect of partially reducing conditions on MMP inhibitory activity and PEGylation.

| Partial Reduction Conditions | Residual Inhibitory Activity | PEGylation |
| --- | --- | --- |
| control | 100% | none |
| 1.25 equiv. TCEP | 90% | ~50% |
| 2.5 equiv. TCEP | 56% | ~80% |
| 5 equiv. TCEP | 0% | ~95% |
| 50 μM DTT | 80% | none |
| 100 μM DTT | 80% | none |
| 200 μM DTT | 70% | none |
| 0.1 mM MEA | 76% | none |
| 1 mM MEA | 62% | ~2% |
| immobilized TCEP, 10′ | 44% | ~90% |
| immobilized TCEP, 30′ | 31.3% | ~90% |
| immobilized DTT, 10′ | 81% | ~2% |
| immobilized DTT, 30′ | 54% | ~5% |
